# Timing of Surgical Axillary Staging and Impact on Technical Success of Immediate Lymphatic Reconstruction Following Axillary Lymph Node Dissection

**DOI:** 10.1245/s10434-026-20142-0

**Published:** 2026-07-01

**Authors:** Helen M. Johnson, Susie X. Sun, David M. Adelman, Edward I. Chang, Solange Cox, Shannon Foreman, Maggie Keener, Lauren Patterson, Simona F. Shaitelman, Kelly K. Hunt, Sarah M. DeSnyder

**Affiliations:** 1 Department of Breast Surgical Oncology, The University of Texas MD Anderson Cancer Center, Houston, TX USA; 2 Department of Plastic Surgery, The University of Texas MD Anderson Cancer Center, Houston, TX USA; 3 Department of Breast Radiation Oncology, The University of Texas MD Anderson Cancer Center, Houston, TX USA

**Keywords:** Immediate lymphatic reconstruction, Lymphovenous bypass, Axillary reverse mapping, Axillary lymph node dissection, Lymphedema, Breast cancer related lymphedema, Breast cancer, Axillary surgery

## Abstract

**Background:**

The impact of sentinel lymph node dissection (SLND) or targeted axillary dissection (TAD) prior to axillary lymph node dissection (ALND) on the technical success of immediate lymphatic reconstruction (ILR) is not well described. We aimed to determine if the technical success of ILR varies by timing of surgical axillary staging prior to ALND.

**Methods:**

Our departmental database was queried to identify patients undergoing ALND from 2020 to 2024. Type and timing of axillary surgical staging and ILR technical details were collected by retrospective chart review.

**Results:**

Among 866 patients undergoing ALND with a plan for ILR, 655 (75.6%) underwent single-stage ALND, 120 (13.9%) underwent SLND/TAD followed by ALND under the same anesthetic, and 91 (10.5%) underwent SLND/TAD followed by ALND at a separate surgery. The overall ILR technical success rate was 93.4% (612/655). Immediate lymphatic reconstruction technical success did not vary significantly between groups (*p* = 0.415). Groups were similar in number of reconstructed lymphatic channels (*p* = 0.173), with a median of 2 (IQR 1–3) channels. The most common reasons for ILR not being performed (*n* = 52) included failure to identify blue lymphatic channels by axillary reverse mapping (*n* = 21) and no suitable veins for the reconstruction (*n* = 11). On multivariable analysis, White race and lower body mass index were significant predictors of ILR technical success.

**Conclusions:**

In patients undergoing ALND, ILR was successfully performed in > 90% of cases, irrespective of concurrent or staged SLND/TAD procedures. Most patients (62.9%) had at least 2 lymphatic channels reconstructed. Deferring decision-making about completion ALND for final pathology from SLND/TAD does not appear to negatively impact technical success of ILR.

Breast cancer-related lymphedema (BCRL) is a chronic condition characterized by swelling, heaviness, discomfort, and decreased range of motion of the upper extremity that affects a significant number of patients during survivorship. Breast cancer-related lymphedema is caused by disruption of the lymphatic system and is a well-described sequela and cause of morbidity of axillary lymph node dissection (ALND).^1–4^ Other BCRL risk factors include greater nodal disease burden,^5^ higher body mass index (BMI),^6,7^ use of axillary radiation,^8,9^ taxane-based chemotherapy,^10^ Black race, and Hispanic ethnicity.^11,12^

Immediate lymphatic reconstruction (ILR) has been shown to decrease the risk of BCRL in patients undergoing ALND.^13–18^ Immediate lymphatic reconstruction involves identification of afferent lymphatic channels draining the upper extremity via axillary reverse mapping (ARM) or other methods and restoration of flow by connecting transected lymphatics to venous tributaries in the axilla. Several ILR techniques have been described including use of microsurgical anastomoses with prophylactic lymphovenous bypass (pLVB) and nonmicrosurgical re-approximation such as Simplified Lymphatic Microsurgical Preventative Healing Approach (S-LYMPHA).^13,19^ Prior work has explored technical factors impacting ILR success and BCRL risk reduction.

The impact of timing of surgical axillary staging prior to ALND on the technical success of ILR is not well described. We aimed to determine if the technical success of ILR varies among patients undergoing single-stage ALND, those undergoing sentinel lymph node dissection (SLND) or targeted axillary dissection (TAD) followed by completion ALND under the same anesthetic, and those undergoing axillary staging followed by ALND performed at two separate surgeries.

## Methods

This retrospective analysis was deemed exempt by the MD Anderson Cancer Center Institutional Review Board (protocol #2024-1733). The Breast Surgical Oncology departmental database was queried to identify patients with breast cancer who underwent ALND from January 1, 2020 through December 31, 2024. Retrospective chart review was performed to collect details regarding the type of axillary surgical staging, timing of axillary surgical procedures, type of ILR, and ILR technical details.

Patients were classified into one of three groups: (1) single-stage ALND; (2) ALND performed immediately after SLND/TAD under the same anesthetic based on intraoperative frozen section pathology results; (3) ALND performed at a second surgery following initial staging SLND/TAD based on permanent pathology results.

Axillary reverse mapping was performed by the breast surgical oncologist with injection of vital blue dye into the upper arm and preservation of blue ARM lymphatic channels during ALND.^20^ A lazy S axillary incision is used when ILR is planned, except in cases involving a total simple mastectomy with sufficient exposure for ILR through the mastectomy incision. ILR techniques were classified as follows: pLVB was defined as a microsurgical procedure performed by a plastic and reconstructive surgeon in which a transected ARM lymphatic channel was connected to an adjacent vein using an operative microscope,^17^ and nonmicrosurgical ILR was defined as a procedure performed by a breast surgical oncologist following ARM without microvascular anastomosis. Nonmicrosurgical ILR procedures included re-approximation of a transected blue arm lymphatic channel to a nearby vein (S-LYMPHA) or to an efferent lymphatic channel.^19,21^

Immediate lymphatic reconstruction technical success was defined and measured by the proportion of patients in whom ILR was planned and completed, as well as the number of reconstructed lymphatic channels.

Patient demographics and clinicopathologic characteristics were reported using descriptive statistics. Chi-square and ANOVA tests were performed to compare differences by ILR technical success. Multivariable logistic regression model was fitted to determine predictors of ILR technical success while controlling for potential confounders. Potential covariates for the multivariable model were selected based on clinical plausibility. Backwards elimination was then performed by using a retention threshold of *p* < 0.15 and clinical significance. Covariate retention was determined based on each covariate’s adjusted association with ILR technical success in the multivariable model rather than its univariate association. Two-tailed *p* values < 0.05 were considered statistically significant, and all statistical analyses were performing by using STATA version 18.3 (StataCorp LP, College Station, TX).

## Results

During the study period, 1887 patients with breast cancer underwent ALND, and ILR was planned in 866. Demographics and clinical characteristics of the patients planned for ILR are presented in Table 1. Most patients (655, 75.6%) underwent single-stage ALND, whereas 120 (13.9%) underwent concurrent SLND/TAD and ALND under the same anesthetic, and 91 (10.5%) underwent staged SLND/TAD followed by ALND at a separate surgery. Whether ILR was planned or not varied significantly between groups (*p* < 0.0001), with higher rates of planned ILR among patients undergoing single-stage ALND.

**Table 1:** Baseline characteristics of patients undergoing axillary lymph node dissection (ALND) with a plan for immediate lymphatic reconstruction (ILR), overall and by timing of surgical axillary staging prior to ALND

	Overall (*n* = 866)	Single-Stage ALND (*n* = 655)	Concurrent SLND/TAD and ALND (*n* = 120)	Staged SLND/TAD Followed by ALND (*n* = 91)	*p*
*Age*					0.075
≤40	18.9% (164)	17.7% (116)	20% (24)	26.4% (24)	
41–50	24.5% (212)	23.5% (154)	30.8% (37)	23.1% (21)	
51–60	26.2% (227)	26% (170)	28.3% (34)	25.3% (23)	
> 60	30.4% (263)	32.8% (215)	20.8% (25)	25.3% (23)	
*Race*					0.632
Asian	8.2% (71)	8.4% (55)	10% (12)	4.4% (4)	
Black	11.9% (103)	12.1% (79)	13.3% (16)	8.8% (8)	
Other	7% (61)	6.4% (42)	8.3% (10)	9.9% (9)	
White	72.2% (625)	72.4% (474)	67.5% (81)	76.9% (70)	
Unknown	0.7% (6)	0.8% (5)	0.8% (1)	0% (0)	
*Ethnicity*					0.288
Hispanic	17% (147)	16.8% (110)	15.8% (19)	19.8% (18)	
Non-Hispanic	80.9% (701)	81.2% (532)	80% (96)	80.2% (73)	
Unknown	2.1% (18)	2% (13)	4.2% (5)	0% (0)	
*Body mass index (kg/m*^*2*^*)*^a^					0.354
Median (IQR)	27 (23–32)	27 (23–32)	28.5 (25–32.5)	26 (23–30.5)	
*Clinical T category*					< 0.0001
T0/Tis	5.9% (51)	6.6% (43)	4.2% (5)	3.3% (3)	
T1	14.5% (126)	12.2% (80)	15.8% (19)	29.7% (27)	
T2	32.9% (285)	29.8% (195)	45.8% (55)	38.5% (35)	
T3	24% (208)	25.2% (165)	17.5% (21)	24.2% (22)	
T4a-c	8.7% (75)	10.1% (66)	5% (6)	3.3% (3)	
T4d	14% (121)	16.2% (106)	11.7% (14)	1.1% (1)	
*Clinical N category*					< 0.0001
N0	10.3% (89)	4.9% (32)	6.7% (8)	53.8% (49)	
N1	55.8% (483)	55.9% (366)	68.3% (82)	38.5% (35)	
N2	6.4% (55)	7.3% (48)	2.5% (3)	4.4% (4)	
N3	27.5% (238)	31.9% (209)	22.5% (27)	2.2% (2)	
Unknown	0.1% (1)	0% (0)	0% (0)	1.1% (1)	
Preoperative chemotherapy					< 0.0001
Yes	79.2% (686)	85% (557)	67.5% (81)	52.7% (48)	
No	2.8% (24)	16.4% (108)	32.5% (39)	47.3% (43)	
*Preoperative radiation*					0.857
Yes	2.8% (24)	2.6% (17)	3.3% (4)	3.3% (3)	
No	97.2% (842)	97.4% (638)	96.7% (116)	96.7% (88)	
*Axillary recurrence with history of prior axillary surgery*					0.010
Yes	4.7% (41)	6% (39)	1.7% (2)	0% (0)	
No	95.3% (825)	94% (616)	98.3% (118)	100% (91)	

*SLND* sentinel lymph node dissection; *TAD* targeted axillary dissection; *IQR* interquartile range

^*^Body mass index missing for 91 patients

Groups were similar with respect to age, race, ethnicity, BMI, or receipt of preoperative radiation (Table 1). Groups differed significantly by clinical T category (*p* < 0.0001), with higher rates of single-stage ALND among patients with cT4 disease and more staged axillary procedures among patients with cT1 disease. Groups also differed by clinical N category (*p* < 0.0001), with higher rates of single-stage ALND among patients with cN3 disease and more staged axillary procedures among patients with cN0 disease. Compared with patients undergoing SLND/TAD prior to ALND, patients undergoing single-stage ALND had higher rates of receipt of preoperative systemic therapy (*p* < 0.0001). In addition, this group had a higher proportion of patients who underwent ALND for axillary recurrence, with a history of prior axillary surgery (*p* = 0.01).

Immediate lymphatic reconstruction was successfully performed in 814 of 866 cases (success rate 94%). Technical success of ILR did not vary significantly between groups (*p* = 0.415, Table 2). Additionally, the number of reconstructed lymphatic channels did not vary significantly between groups (*p* = 0.173, Table 2), with a median of 2 (IQR 1–3) channels identified with ARM.

**Table 2:** Technical success of immediate lymphatic reconstruction (ILR) by timing of surgical axillary staging prior to axillary lymph node dissection (ALND)

	Single-Stage ALND (*n* = 655)	Concurrent SLND/TAD and ALND (*n* = 120)	Staged SLND/TAD Followed by ALND (*n* = 91)	*p*
*ILR successfully performed*				
Yes	612 (93.4%)	114 (95%)	88 (96.7%)	0.415
No	43 (6.6%)	6 (5%)	3 (3.3%)	
No. reconstructed channels, median (IQR)	2 (1–3)	2 (1–3)	2 (1–3)	0.173
*Type of ILR planned*				
Lymphovenous bypass	592 (90.4%)	95 (79.2%)	78 (85.7%)	0.001
Nonmicrosurgical ILR	63 (9.6%)	25 (20.8%)	13 (14.3%)	
*Technical success rate, by procedure*				
Lymphovenous bypass	555/592 (93.8%)	93/95 (97.9%)	76/78 (97.4%)	0.005
Nonmicrosurgical ILR	57/63 (90.5%)	21/25 (84%)	12/13 (92.3%)	

*SLND* sentinel lymph node dissection; *TAD* targeted axillary dissection; *IQR* interquartile range

Overall, the most common ILR technique was pLVB, with 765 of 866 (88.3%) patients planned for this procedure. In contrast, 101 patients (11.8%) were planned for nonmicrosurgical ILR performed by the breast surgical oncologist. Planned ILR technique differed significantly between groups (*p* = 0.001, Table 2), with a greater proportion of patients undergoing single-stage ALND planned for pLVB. The highest rates of planned non-microsurgical ILR (20.8%) were observed in patients undergoing concurrent SLND/TAD and ALND and the lowest rates (9.6%) in those undergoing single-stage ALND (Table 2). Overall, technical successful of ILR was higher for pLVB compared with nonmicrosurgical ILR: 94.6% (724/765) versus 89.1% (90/101). Technical success of ILR by technique varied significantly between groups (*p* = 0.005, Table 2), with the lowest success rates observed for patients undergoing concurrent SLND/TAD and ALND planned for non-microsurgical ILR (84%) and the highest seen for concurrent SLND/TAD and ALND planned for pLVB (97.9%).

Immediate lymphatic reconstruction was planned but unable to be performed in 52 patients. Reasons for failure to perform ILR are presented in Table 3. The most common contributing factor was inability to identify lymphatic channels by axillary reverse mapping (*n* = 21, 40.4%), followed by lack of suitable venous tributaries (*n* = 11, 21.2%).

**Table 3:** Reasons that a planned immediate lymphatic reconstruction was not performed (*n* = 52), overall and by timing of surgical axillary staging prior to ALND

Primary Contributing Factor	Total no. Patients	Single-Sage ALND	Concurrent SLND/TAD and ALND	Staged SLND/TAD Followed by ALND
Failure to identify lymphatic channels by axillary reverse mapping	21	18	2	1
No suitable veins	11	10	0	1
All lymphatic channels intact without disruption	9	6	2	1
Insufficient lymphatic channel caliber	3	3	0	0
Insufficient lymphatic channel length	3	2	1	0
Lymphatic channel-vein caliber mismatch	3	3	0	0
Lymphatic channels disrupted	2	1	1	0

*ALND* axillary lymph node dissection; *SLND* sentinel lymph node dissection; *TAD* targeted axillary dissection

On univariate analysis, significant predictors of technical success of ILR included race (*p* = 0.032) and ethnicity (*p* = 0.016), with the highest success rates observed in White patients and non-Hispanic patients (Table 4). In addition, lower technical success rates were observed in patients with higher BMI (*p* = 0.089) and patients with a diagnosis of axillary recurrence and a history of prior axillary surgery (*p* = 0.087). Technical success of ILR was not significantly associated with age, clinical stage, use of preoperative systemic therapy, or use of preoperative radiation (Table 4).

**Table 4:** Univariate associations between technical success of immediate lymphatic reconstruction (ILR) and baseline characteristics and timing of surgical axillary staging prior to axillary lymph node dissection (ALND)

	ILR Planned and Successfully Performed (*n* = 814)	ILR Planned But not Successfully Performed (*n* = 52)	*p*
*Age (year)*			
≤ 40	19.4% (158)	11.5% (6)	0.577
41–50	24.3% (198)	26.9% (14)	
51–60	26% (212)	28.8% (15)	
> 60	30.2% (246)	32.7% (17)	
*Race*			
Asian	8.1% (66)	9.6% (5)	0.032
Black	11.4% (93)	19.2% (10)	
Other	6.5% (53)	15.4% (8)	
White	73.2% (596)	55.8% (29)	
Unknown	0.7% (6)	0% (0)	
*Ethnicity*			
Hispanic	16.1% (131)	30.8% (16)	0.016
Non-Hispanic	81.7% (665)	69.2% (36)	
Unknown	2.2% (18)	0% (0)	
*Body mass index (kg/m*^*2*^*)*			
Median (IQR)	27 (23–31)	30 (27–36)	0.089
*Clinical T category*			
T0/Tis	5.9% (48)	5.8% (3)	0.981
T1	14.4% (117)	17.3% (9)	
T2	33.2% (270)	28.8% (15)	
T3	24% (195)	25% (13)	
T4a–c	8.7% (71)	7.7% (4)	
T4d	13.9% (113)	15.4% (8)	
*Clinical N category*			
N0	10.4% (85)	7.7% (4)	0.416
N1	56.4% (459)	46.2% (24)	
N2	6.3% (51)	7.7% (4)	
N3	26.8% (218)	38.5% (20)	
Unknown	0.1% (1)	0% (0)	
*Preoperative chemotherapy*			
Yes	79.4% (646)	76.9% (40)	0.674
No	2.7% (22)	3.8% (2)	
*Preoperative radiation*^a^			
Yes	2.7% (22)	3.8% (2)	0.626
No	97.3% (792)	96.2% (50)	
*Axillary recurrence with history of prior axillary surgery*			
Yes	4.4% (36)	9.6% (5)	0.087
No	95.6% (778)	90.4% (47)	

*IQR* interquartile range

^a^Preoperative radiation was delivered on clinical trial and included targeting of the breast and regional nodal basins, including but not limited to levels I–III of the axilla

On multivariable analysis, race and BMI remained significant predictors of technical success of ILR (Table 5). Patients of White race had the highest likelihood of ILR technical success, whereas patients of “Other” race had significantly lower likelihood of ILR technical success (odds ratio [OR] 0.34, 95% confidence interval [CI] 0.15–0.79, *p* = 0.013). Compared with patients with BMI > 31, those with BMI ≤ 23 had significantly higher likelihood of technical success of ILR (OR 3.93, 95% 1.31–11.82, *p* = 0.015).

**Table 5:** Multivariable associations between technical success of immediate lymphatic reconstruction (ILR) and baseline characteristics

	Odds Ratio	95% Confidence Interval	*p*
*Race*			
Asian	0.54	0.20–1.47	0.227
Black	0.54	0.25–1.17	0.117
Other	0.34	0.15–0.79	0.013
White	Reference		
*Body mass index (kg/m*^*2*^*)*			
≤ 23	3.93	1.31–11.82	0.015
24–27	1.39	0.68–2.85	0.362
28–31	1.22	0.58–2.57	0.608
> 31	Reference		
*Axillary recurrence with history of prior axillary surgery*			
Yes	0.44	0.16–1.21	0.111
No	Reference		

## Discussion

In this large cohort of patients undergoing ALND, technical success of ILR was high (> 90%) and was not significantly associated with timing of surgical axillary staging. In addition, the number of reconstructed lymphatic channels (median 2) was similar between patients undergoing single-stage ALND, concurrent SLND/TAD and ALND under the same anesthetic, or staged SLND/TAD followed by ALND. Overall, these data suggest that deferring decision-making about completion ALND to await final surgical pathology from SLND/TAD does not appear to negatively impact the technical success of ILR.

Since 2017, the use of ILR has been increasing in the United States as a preventative strategy for BCRL,^22^ a common morbidity from ALND that decreases the quality of life of breast cancer survivors.^23,24^ A recent meta-analysis of nine prospective studies concluded that ILR significantly lowers the risk of BCRL, with patients undergoing re-approximation of a mean of two lymphatic channels having 69% lower odds of developing BCRL compared with those not undergoing ILR.^25^ Over the past several years at our comprehensive cancer center, approximately half of patients undergoing ALND were offered ILR. During this same time period, advances in the de-escalation of axillary surgery have resulted in fewer patients undergoing single-stage ALND with more being offered SLND^26–28^ or TAD^29,30^ following neoadjuvant chemotherapy. Depending on the pathology results from SLND/TAD, some of these patients undergo completion ALND, either under the same anesthetic if frozen section pathology is utilized or at a second operation if decision-making is deferred to permanent pathology. Violation of tissue planes, scarring, lymphatic disruption, and disruption of venous tributaries may occur during or after SLND/TAD and could impact the technical success of ILR. Even when performed under a single anesthetic, surgical axillary staging prior to ALND may result in more trauma to the lymphatics and veins than single-stage ALND as directed excision of multiple individual nodes requires more extensive dissection and violation of tissue planes. Our study provides the first evidence that undergoing surgical axillary staging prior to ALND does not significantly impact the technical success of ILR. Offering de-escalation of axillary surgery when oncologically appropriate does not appear to compromise future ability to perform ILR in patients who may require completion ALND and benefit from BCRL risk-reduction.

Prior studies exploring factors associated with failure to successfully perform ILR have focused primarily on technical factors. Two prior studies reported the most common reason for failure to perform ILR was lack of an appropriate venous tributary.^31,32^ Conversely, others have reported that the inability to identify lymphatic channels was the primary reason for failure to perform ILR, followed by lack of suitable veins.^15^ This is consistent with our findings, with 40% of the failed ILR attempts in our cohort resulting from inability to identify lymphatic channels following ARM and 21% owing to inadequate recipient veins. Our study offers additional unique insights into technical contributors to failure to successfully perform ILR, including short lymphatic channel length, small lymphatic channel caliber, and lymphatic channel-vein caliber mismatch. Furthermore, our observation that prior axillary surgery in patients undergoing ALND for axillary recurrence did not impact the likelihood of technical success of ILR suggests that concerns about the impact of scarring in the axilla may be overstated.

We report several notable associations between demographic factors and technical success of ILR. A prior study reported lower odds of ILR success among patients with higher BMI, as well as among current and former smokers.^31^ Similarly, we also observed a lower rate of successful completion of ILR among patients with BMI > 31. Unfortunately, these patients are also at increased risk of BCRL^6,7^ and therefore may derive a greater magnitude of benefit from ILR if able to be performed. In addition to BMI, we observed a significant association between race and technical success of ILR, with White patients experiencing the highest likelihood of technical success. As patients of Black race have been shown to have higher rates of BCRL than their White counterparts, limitations in ILR performance may be particularly impactful.^11,12^ Further research is needed to determine whether technical modifications may increase the likelihood of successful completion of ILR in these high-risk populations.

Interestingly, the overall high rate of technical success of ILR of 94% in our cohort is superior to that of previously reported cohorts. Feldman and colleagues reported the first experience in the United States with ILR in 2015, with a success rate of 73% (27/37 cases) using the LYMPHA technique.^15^ Subsequently, another report from a different academic medical center utilizing pLVB from 2016 to 2020 had greater technical success, with a rate of 84.9% (128/186 cases).^16^ Investigators from a community hospital reported their implementation of an ILR program, with a success rate of 76.7% (69/90 patients) from 2019 to 2021.^31^ This is comparable to the success rate observed in a randomized control trial evaluating the efficacy of pLVB in preventing BCRL.^17^ In an interim analysis from this trial, Coriddi and colleagues stated that 44 of 196 patients enrolled from 2020–2023 were deemed ineligible due to lack of veins or lack of lymphatics for bypass, corresponding to a technical success rate of 77.6%.^17^ A higher success rate of 87.8% was reported by Jakub and colleagues in a prospective study of 131 patients planned for ILR utilizing a combination of techniques from 2018 to 2023 at the Mayo Clinic.^33^ Our technical success rate of 94% may be attributable to several factors, including a more contemporary cohort treated from 2020 to 2024 after maturation of the institutional ILR program, high-volume practice, availability of surgical subspecialists with expertise in microvascular surgery, and high utilization of pLVB.

At our institution, ILR is most commonly performed by plastic and reconstructive surgeons using pLVB technique, while some patients undergo non-microsurgical ILR by the breast surgical oncologist. There were a total of 33 plastic and reconstructive surgeons and 27 breast surgical oncologists who performed these procedures on the patients analyzed in this study. We observed a higher technical success rate for completion of ILR with microvascular anastomosis compared with nonmicrosurgical ILR (94.6% vs. 89.1%). However, when ILR was able to be performed, the number of reconstructed channels identified by ARM was similar regardless of reconstructive technique. The aforementioned prospective trial at Mayo Clinic utilized multiple ILR techniques, and the authors noted a trend toward lower BCRL rates for patients undergoing microsurgical ILR performed by a plastic surgeon compared with those undergoing nonmicrosurgical ILR performed by a breast surgical oncologist (*p* = 0.065). While the investigators did not report any intraoperative failures, our results are broadly similar. Additional studies are needed to clarify the impact of surgical technique and surgeon experience on successful completion and efficacy of ILR, including both short-term outcomes, such as technical completion and number of reconstructed channels and long-term outcomes, such as BCRL and quality of life. These data may also help to inform decisions about the value of these procedures, as reimbursement for microsurgical ILR is currently quite low considering the necessary investment in technical training, equipment costs, and operating room time.^34^

Our study is limited by its retrospective nature as well as a low number of events (unsuccessful ILR attempts) restricting the number of covariates we were able to analyze in multivariable analyses. In addition, we were unable to explore associations between other potentially interesting variables, such as the impact of smoking due to significant missing data. Results from our cohort of patients treated at a high-volume comprehensive cancer center with a large number of subspecialty-trained microvascular surgeons may not be generalizable to the broader national population. Finally, we are unable to comment on the efficacy of ILR with respect to BCRL risk-reduction and quality of life outcomes as this study focuses solely on the technical success of ILR.

## Conclusions

This study provides the first evidence that concurrent or recent surgical axillary staging prior to ALND does not appear to negatively impact the technical success of ILR. Awaiting final surgical pathology from SLND or TAD procedures to make decisions about the need for additional axillary surgery is unlikely to compromise future ability to perform ILR in patients who ultimately require completion ALND and may benefit from BCRL risk reduction.
